# Structural Optimization Design of Dual Robot Gripper Unloading Device Based on Intelligent Optimization Algorithms and Generative Design

**DOI:** 10.3390/s23198298

**Published:** 2023-10-07

**Authors:** Jiguang Jia, Xuan Sun, Ting Liu, Jiazhi Tang, Jiabing Wang, Xianxuan Hu

**Affiliations:** 1 College of Mechanic and Control Engineering, Guilin University of Technology, Guilin 541004, China; jjg@glut.edu.cn (J.J.); 2120211165@glut.edu.cn (T.L.); azureaurora284@gmail.com (J.T.); 15832703687@163.com (J.W.); huhuxianxuan@outlook.com (X.H.); 2 Key Laboratory of Advanced Manufacturing and Automation Technology, Education Department of Guangxi Zhuang Autonomous Region, Guilin University of Technology, Guilin 541006, China

**Keywords:** NSGA-III algorithm, design for generative theory, comprehensive optimization design methodology, meta-model, principle of cooperative equilibrium

## Abstract

The main aim of this paper is to explore new approaches to structural design and to solve the problem of lightweight design of structures involving multivariable and multi-objectives. An integrated optimization design methodology is proposed by combining intelligent optimization algorithms with generative design. Firstly, the meta-model is established to explore the relationship between design variables, quality, strain energy, and inherent energy. Then, employing the Non-dominated Sorting Genetic Algorithm III (NSGA-III), the optimal frameworks of the structure are sought within the entire design space. Immediately following, a structure is rebuilt based on the principle of cooperative equilibrium. Furthermore, the rebuilt structure is integrated into a generative design, enabling automatic iteration by controlling the initial parameter set. The quality and rigidity of the structure under different reconstructions are evaluated, resulting in solution generation for structural optimization. Finally, the optimal structure obtained is validated. Research outcomes indicate that the quality of structures generated through the comprehensive optimization method is reduced by 27%, and the inherent energy increases by 0.95 times. Moreover, the overall structural deformation is less than 0.003 mm, with a maximum stress of 3.2 MPa—significantly lower than the yield strength and meeting industrial usage standards. A qualitative study and analysis of the experimental results substantiate the superiority of the proposed methodology for optimized structural design.

## 1. Introduction

In recent years, robot grippers have found widespread application across industries such as manufacturing, logistics, and healthcare [1,2,3]. An increasing number of sectors are using robot grippers to execute tasks and have also devised equipment compatible with these grippers [4,5]. Recently, a dual robot gripper unloading device has been developed to meet the demands of the logistics sector. To devise a structure for an unloading device that coordinates with dual robot grippers, this study presents a comprehensive optimization design approach based on the intelligent optimization algorithm NSGA-III and generative design theory, using this method to optimize the structure of the unloading device with dual robot grippers.

In the structural design of the dual robot gripper unloading device, lightweight design principles are considered while ensuring that the structure’s performance meets the required specifications. There are multiple goals involved. When addressing multi-objective optimization problems [6], conventional optimization employs transformation methods to reframe multi-objective problems into single-objective ones for resolution. However, this approach can no longer handle multi-objective optimization problems with complex constraints in engineering. In multi-objective optimization, experts have explored and studied numerous theories and design approaches for intelligent optimization algorithms to tackle this issue. Among them, the Genetic Algorithm [7], AOA Algorithm [8], PSO Algorithm [9], and SCA Algorithm [10] have been applied to the study of multi-objective optimization problems. The research findings indicate that genetic algorithms possess straightforward principles and exceptional global search capabilities, leading many scholars to employ this method in addressing multi-objective optimization problems. Zhang et al. [11] utilized the NSGA-II algorithm to optimize the connectivity structure, achieving excellent structural performance. For foldable structures, Kaveh et al. [12] employed the NSGA-II algorithm to reduce the weight and volume of the structure. Regarding heat exchanger parameter optimization, Guan et al. [13] used the MOGA design method. The above optimization methods have shown excellent performance in handling low-latitude, multi-objective problems. However, when it comes to high-latitude multi-objective problems related to unloading device structures, a challenge arises in achieving a low convergence rate of results. Addressing this issue, Jain et al. [14] proposed a non-dominated sorting optimization algorithm utilizing reference points, namely NSGA-III, which effectively resolved high-latitude multi-objective optimization problems. In subsequent studies, Qu et al. [15] employed the NSGA-III algorithm to obtain optimal design solutions for a robot suspension system. Li, B. et al. [16], in their endeavor to enhance optimizer efficiency, confirmed the superiority of NSGA-III in tackling multi-objective optimization problems. Additionally, Li, X. et al. [17] employed the NSGA-III method for multi-objective optimization design. Zhang P. [18] used the NSGA-III algorithm to optimize the main dimensions of the articulated problem to obtain a Pareto optimal solution set. Xu J. et al. [19] performed the geometric optimization of rotor blades based on the NSGA-III algorithm to achieve maximum power and minimum hydrodynamic forces. The results of these experts show that the NSGA-III algorithm performs well in optimizing geometries. Hence, in this paper, NSGA-III is adopted for optimization design to attain the optimal overall framework of the structure, laying the foundation for subsequent generative design.

Generative design technology can rapidly and automatically explore solution spaces [20,21]. Once solutions are obtained, they are presented to design engineers visually, allowing for selection and further in-depth design. Furthermore, generative design is a mass-customization technique that can generate many solutions for the same problem by adjusting a preset initial parameter set. This method employs the principles of topology optimization to generate design configurations. Topology optimization is rooted in applying algorithms to design the material distribution within an optimization region based on given design requirements, thereby generating structures that meet the specified criteria. Topology optimization is widely applied across various fields. Lakshmi Srinivas et al. [22] employed topology optimization techniques to reduce the deflection and stress of structures. Srinivas, G.L. et al. [23] utilized topology optimization to design the structural shape of lightweight mechanical arm links. Alkalla M.G. and Liang M. [24,25], among others, employed topology optimization to alter the frameworks and shapes of robot structures. Lakshmi Srinivas et al. [26] improved energy efficiency by defining the design space for topology materials, thus reducing mass and inertia effects.

The aforementioned research results indicate that topology optimization can explore the space of topology solutions by altering input variables. Therefore, generative design can employ topology optimization to create the form of solutions, thereby obtaining optimized structures. Generally, the process of generative design is divided into the following stages: first, problem definition, followed by conceptual design, and finally, detailed structural design [27]. This process requires manual execution and can be iterated to fulfill design requirements. Solutions in generative design are not generated based on the engineers’ inspiration, but rather are calculated and generated using algorithms. These algorithms define the rules for generating structures rather than specific structures [28]. In the initial stages of generative design, solutions with poor performance can be rapidly evaluated, saving time during the structural generation process [29]. Implementing generative design requires using an expert system, which is a tool with specific algorithmic structures that can make decisions comparable to those of experts under well-defined premises [30]. Design structures adhering to explicitly defined parameters are eventually generated through the generative design process. Generative design technology has reached a relatively mature state and is applied in various industries, such as robot structures [31], architectural design [32], and vehicle structure design [33]. In addition, Wang H. et al. [34] applied generative design to practical engineering and successfully designed a treelike column structural joint with superior performance through generative techniques. Jia et al. [35] combined generative design and additive manufacturing techniques for use in structural research in aerospace. Based on the findings of the experts, it can be seen that generative design techniques can be used in conjunction with other techniques to create structures with superior performance.

Based on the studies mentioned above, this paper proposes an integrated design methodology to solve the problem of lightweight design of structures involving multivariable and multi-objective The proposed method can accurately search the solution space of design variables, optimize the overall structural dimensions, and obtain multiple design structures that satisfy different objectives. In addition, the method can generate the required geometries and structural solutions in a shorter time. First, a CCD experimental design was used to obtain various components by orthogonal combinations of dimensional parameters. Then, finite element software was used to iteratively calculate different components and establish a meta-model to analyze the relationship between variables. The overall structural framework is optimized based on the NSGA-III algorithm. Finally, the structure obtained from the integrated optimized design was verified, and the results showed that the method was effective.

## 2. Methodology of the Study

**Figure 1:**
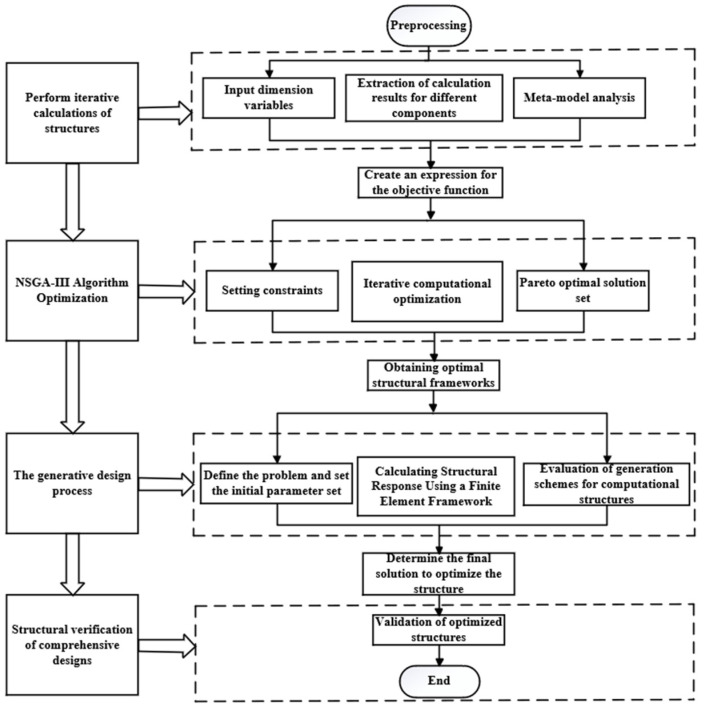
Methodology of the study.

This study aims to integrate an intelligent optimization algorithm (NSGA-III) with a generative design to achieve comprehensive optimization. To accomplish this goal, this research primitively employs FEA and RSM software to establish meta-models, analyzing the interrelationships among various variables. Establish a target function through nonlinear regression analysis to model the relationships between design variables, quality, strain energy, and inherent energy. Subsequently, utilize the iterative optimization mechanism of the NSGA3 algorithm to solve the target function and obtain the Pareto optimal solution set. Apply the principle of cooperative equilibrium to obtain the optimal structural framework. Embed this structural framework into generative design for comprehensive optimization. Finally, based on the ultimate solution obtained, reconstruct the structure and complete the structural simulation validation. The flow of the method is shown in Figure 1.

### 2.1. NSGA-III Algorithm Optimization Process

The structural optimization problem in this paper exhibits complexity with multiple parameters, objectives, and constraints. Therefore, the NSGA-III algorithm is employed for optimization design to attain the Pareto optimal solution set that satisfies multiple objective constraints. Although NSGA-III has the same basic framework as NSGA-II, it outperforms NSGA-II in handling high-latitude problems. As the number of non-dominated solutions increases, NSGA-II performs poorly in determining the tradeoff surface. Additionally, NSGA-II relies on crowding distance ranking [36,37]. NSGA-III employs a predefined set of reference points to maintain population diversity, effectively addressing the low convergence rate issue [38]. The total number of reference points R is as follows:

(1) $$\left\{\begin{array}{l} R=\left(\begin{array}{c} M+n-1\\ n \end{array}\right)\\ R=\frac{\left(M+n-1\right)!}{n!\left(M-1\right)!} \end{array}\right.$$

where n denotes partitioning. In the pre-adaptive normalization stage of population members, an ideal point $\bar{z}=\left(z_{1}{}^{\min},z_{1}{}^{\min},z_{1}{}^{\min},\ldots ,z_{M}{}^{\min}\right)$ is constructed to determine the ideal point of the population $S_{t}$. Then, ASF is employed with the weight vector W as the axis direction to search for extremal values $z^{j,\max}$.

(2) $$\left\{\begin{array}{l} z^{j,\max}=s:argmin_{s\in S_{t}}ASF(s,w^{j})\\ w^{j}=\left(l,\ldots l\right),l\\ l=10^{-6},w_{j}{}^{j}=1 \end{array}\right.$$

In the above equation, s denotes the reference point. The objective function can be normalized as

(3) $$\left\{\begin{array}{l} f_{i}{}^{n}\left(x\right)=\frac{f_{i}{}^{\prime }\left(x\right)}{a_{i}-z_{i}{}^{\min}}\\ f_{i}{}^{n}\left(x\right)=\frac{f_{i}\left(x\right)-z_{i}{}^{\min}}{a_{i}-z_{i}{}^{\min}},i=1,2,3,\ldots ,M \end{array}\right.$$

In the above equation, $f_{i}{}^{\prime }$ is the target direction, $a_{i}$ is the linear hyperplane intercept, and M is the polar vector. After adaptive normalization, each population member is associated with a reference point to calculate the Euclidean distance.

(4) $$\left\{\begin{array}{l} W=z\left(z\in Z^{T}\right)\\ q^{⊥}\left(s,w\right)=\left\|s-\frac{w^{T}sw}{{\left\|w\right\|}^{2}}\right\| \end{array}\right.$$

**Figure 2:**
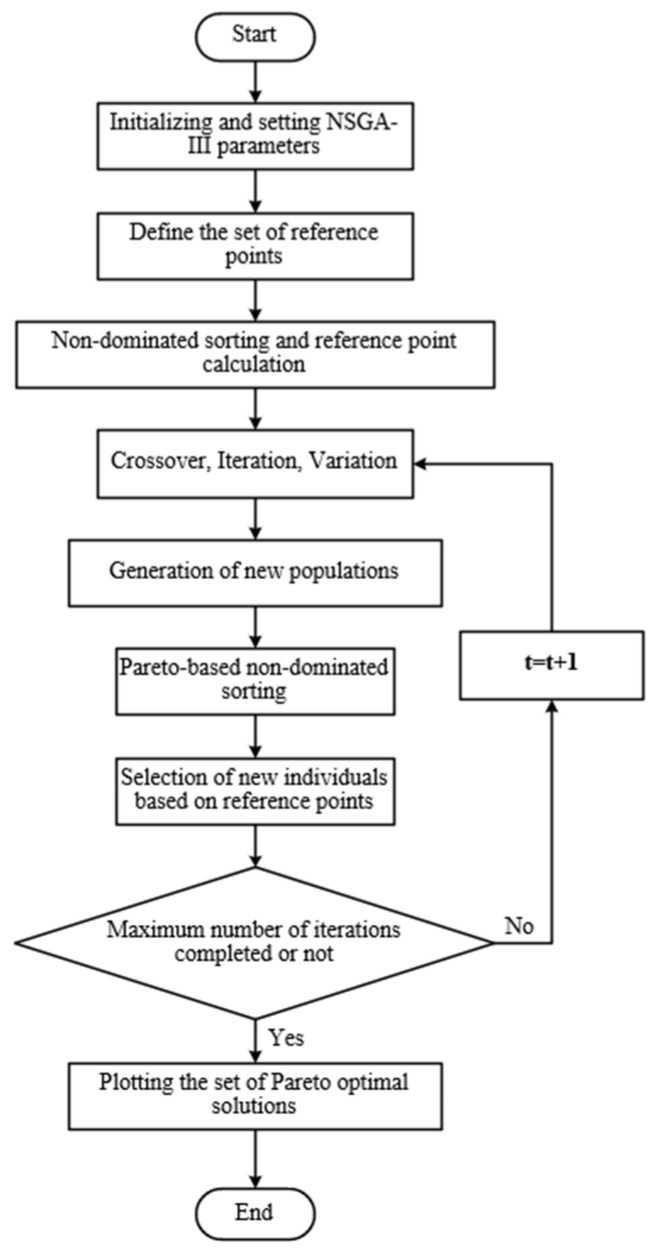
Iterative computation process of NSGA-III algorithm.

In the above equation, the distance between the ideal value and the reference point $\left(s\right)$ is obtained as

(5) $$\left\{\begin{array}{l} d\left(s\right)=w:argmin_{w\in Z^{T}}q^{⊥}\left(s,w\right)\\ q\left(s\right)=q^{⊥}\left(s,d\left(s\right)\right) \end{array}\right.$$

where $q\left(s\right)$ calculates the congestion vector n at the reference point, and the Pareto optimal solution set of the objective function can be fully obtained by NSGA-III. The specific algorithm flow is shown in Figure 2.

### 2.2. Generative Design Theory

Generative design is an optimization method that achieves the automatic construction of structures based on a set of rules or algorithms. The optimization process can be described using the following principles: (1) Define an initial parameter set within the system to generate every potential design. (2) Determine system objective functions that minimize or maximize function values. (3) Establish a set of functions to constrain the system’s boundaries [31].

(6) $$\left\{\begin{array}{l} f_{a}\left(x\right),\left(a=1,2,3,\ldots ,N\right)\\ P_{b}\left(x\right)=0,\left(b=1,2,3,\ldots ,B\right)\\ O_{c}\left(x\right)\leq 0,\left(c=1,2,3,\ldots ,C\right) \end{array}\right.$$

The generative design methodology should be combined with appropriate optimization strategies to assess the quality of the designed structure. When performing numerical simulations in generative design, the key lies in formulating the mathematical expressions of the problem, as shown in Equation (7).

(7) $$Y=H\left(M/Z\right)$$

In the above equation, H is the operator that models M on Z data, where M should be defined as having a flexible structure that can be computationally generated by changing the attributes of a set of parts.

(8) $$M=\langle S,E\left\{a_{1:\left|A\right|}\right\}\rangle$$

where $S,E\left\{a_{1:\left|A\right|}\right\}$ denotes a set of components and $a_{1:\left|A\right|}$ parameter sets of functional characteristics S, and E denotes the relationship between $S,\left\{a_{1:\left|A\right|}\right\}$. The mathematical model constructed by the theory of design for generative can be formulated as an optimization task, as in Equation (9) below.

(9) $$JP^{\max}\left(M*\right)=\underset{M}{\max}f_{P}\left(M/U,T_{1}\leq T_{g}\right),M=\left\{M_{p}\right\}$$

where $f_{P}$ is the fitting function, $JP^{\max}$ is the maximum value of the fit function, and M is the space in which the structure may be generated. For U, it is the space of the actual calculation process. $T_{1}$ is the standard set, and $T_{g}$ is the near-limit value of the optimized structure. The comprehensive optimization design discussed in this paper aims to achieve a lightweight structure through optimization, ensuring sufficient structural stiffness with minimal material usage. For this purpose, the optimization goals are set to minimize volume and compliance. The structural optimization problem addressed in this paper can be formulated as follows:

(10) $$Min:\left\{\begin{array}{l} f_{1}=V/V_{\max}\\ f_{2}=u^{T}Ku \end{array}\right.$$

where $V_{\max}$ is the volume of the design region of the structure, and V is the volume generated by the final optimization. u and K denote the displacement vector and global stiffness matrix.

## 3. Structure Description of Double Robot Gripper Unloading Device

**Figure 3:**
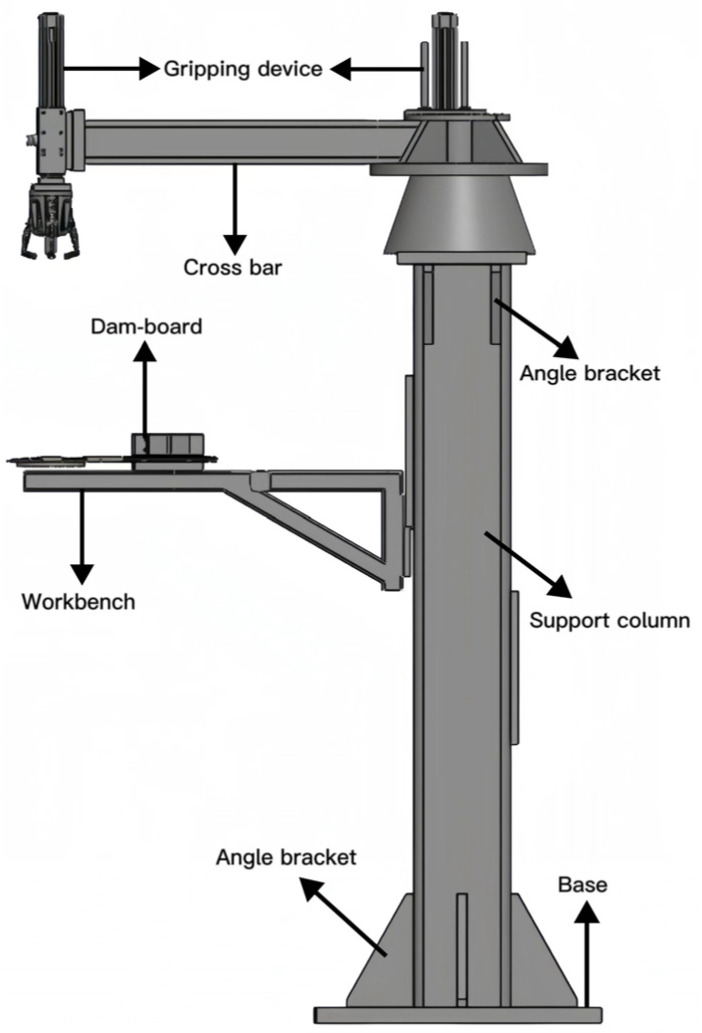
Structure of the dual robot gripper unloading device.

The dual robot gripper device studied in this paper is used for material unloading within a factory. The device is shown in Figure 3 below. In practice, the two robot grippers can rotate 360 degrees to grasp the material. The device links the base and upper components through supporting column structures. The supporting column structure influences the stability of the gripping device and the rotational part. Furthermore, the forces of the entire structure are predominantly concentrated on the supporting columns. Due to the supporting column structure’s pivotal role within the apparatus, this study focuses on optimizing the supporting column structure.

**Figure 4:**
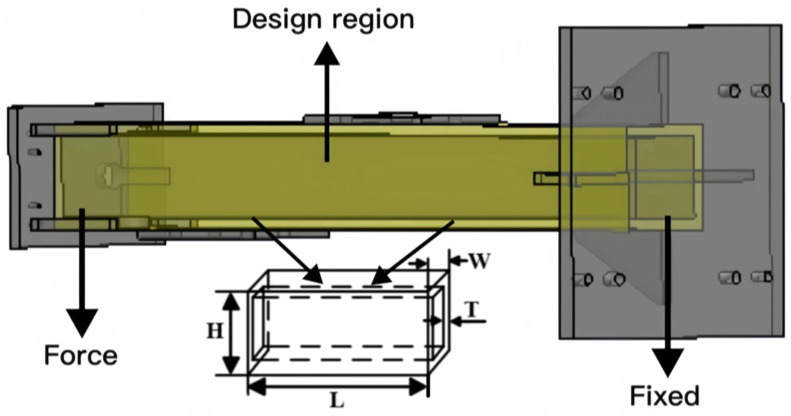
Support column structure.

The ends of the structure, along with the bracing plates and fixed plates, are excluded from the design. The fillet and small hole features of the supporting column components were neglected. This paper attempts to redesign the supporting column structure using the proposed comprehensive optimization method. The goal is to achieve a low structural weight while simultaneously enhancing structural stiffness. The design region is shown in Figure 4.

### 3.1. Design of Experiments

A central composite design (CCD) [39,40] was employed to explore the entire design space. The core idea of CCD is to select a central point and a series of factor variable levels and to construct a set of experimental points to cover the range of variable values. The design region for the supporting column structure is cuboid in shape, with the length (L), width (W), height (H), and thickness (T) as design variables. Ensuring normal usage conditions, the design variable ranges for the supporting column component are L = (180–220 mm), W = (162–198 mm), H = (1244.7–1521.3 mm), T = (18–22 mm). The CCD experimental design (Table 1) obtained 25 experimental groups for the supporting column component.

**Figure 5:**
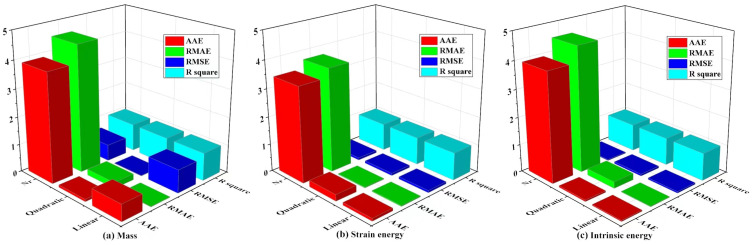
Fitting accuracy of the model.

This research primarily investigates the relationship between design variables and response variables, with response variables encompassing the structure’s mass, strain energy, and inherent energy. Among these, a lower strain energy effectively absorbs the energy generated by external loads, enhancing structural stability and flexural stiffness. Conversely, a higher inherent energy reduces structural vibrations. To comprehensively explore the relationships between structural mass, strain energy, inherent energy, and design variables, the RSM [41] was employed to establish the mapping between design variables and response variables. Concurrently, to ensure accuracy, linear, quadratic polynomial [41] (as shown in Equation (11)), and non-parametric regression [42] (as shown in Equation (12)) models were evaluated to achieve optimal fitting performance. The fitting precision of the design variables, mass, strain energy, and the inherent energy of the supporting column structure is illustrated in Figure 5.

(11) $$f(N)=\alpha_{0}+\sum_{i=1}^{t}b_{i}n_{i}+\sum_{i=1}^{t}c_{i}n_{i}{}^{2}+\sum_{i=1}^{t}\sum_{i<r}^{t}d_{ir}n_{i}n_{r}$$

where $n_{i}$ and $n_{r}$ are the design variables in N. $n_{i}{}^{2}$ is the second-order nonlinearity. $n_{i}n_{r}$ is the interaction between the parameters, and $\alpha_{0}$, $b_{i}$, $c_{i}$, and $d_{ir}$ are the regression coefficient.

(12) $$\left\{\begin{array}{l} Y\leq W,X>b,X=\left\{x_{1},x_{2},\ldots ,x_{M}\right\}\\ Y=\sum_{i=1}^{Q}\left(A_{i}-A_{i}{}^{*}\right)K\left(\vec{X_{i}},\vec{X}\right)+b \end{array}\right.$$

where W is the weighted variable, $\left\{x_{1},x_{2},\ldots ,x_{M}\right\}$ is the input variable, $K\left(\vec{X_{i}},\vec{X}\right)$ is the mapping, and $A_{i},A_{i}{}^{*}$ is the Lagrange multiplier.

During the fitting process, different mathematical models exhibit different fitting accuracies. By evaluating the Absolute Average Error (AAE) and Relative Mean Absolute Error (RMAE), this study found that the values from non-parametric regression significantly exceeded the baseline value of 1, indicating an absolute difference. In general, the smaller the values of RMSE and RMAE, the smaller the prediction error of the model and the higher the fitting accuracy. The comparative results for the maximum RMSE and RMAE in linear, quadratic, and non-parametric regression models are as follows:

(1) The maximum RMSE and RMAE of the nonparametric regression model are 0.60 and 4.54, respectively, and these values are relatively high, indicating that the model has a large prediction error and low fitting accuracy.

(2) The maximum RMSE and RMAE of the linear model are 0.84 and 0.019, respectively, and although the RMSE is slightly higher, the RMAE value is lower, indicating that the fitting accuracy of the linear model is relatively good.

(3) The maximum RMSE and RMAE for the quadratic polynomial model are 0.016 and 0.24, respectively. These values are relatively small, indicating a high level of fitting accuracy for the quadratic polynomial model. Furthermore, the R-squared values for both the linear and quadratic polynomial models are greater than 0.9. This shows that the linear and quadratic polynomials are fitted with high accuracy and can effectively demonstrate the relationship between the design and response variables.

### 3.2. Establishment of Meta-Model

**Figure 6:**
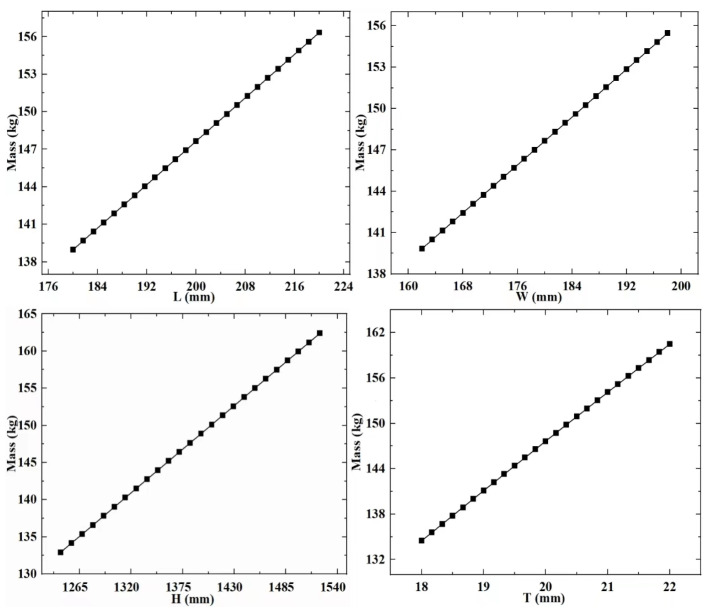
Meta-model a.

**Figure 7:**
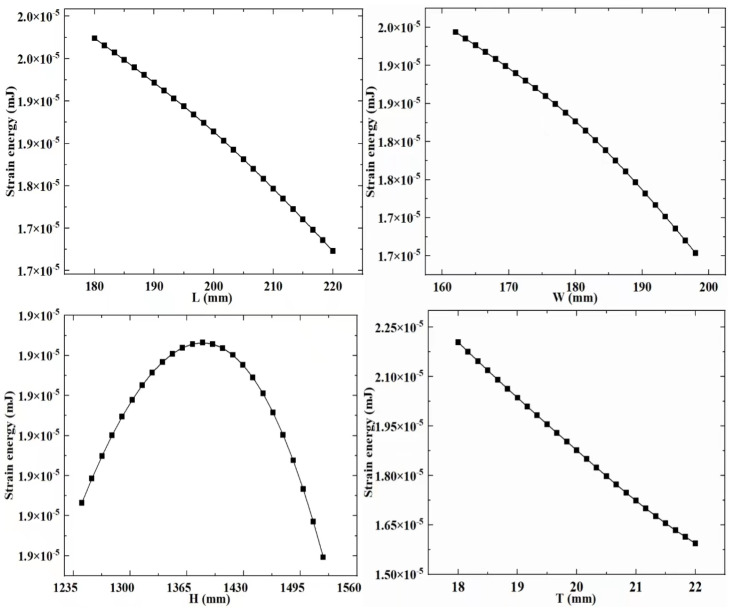
Meta-model b.

**Figure 8:**
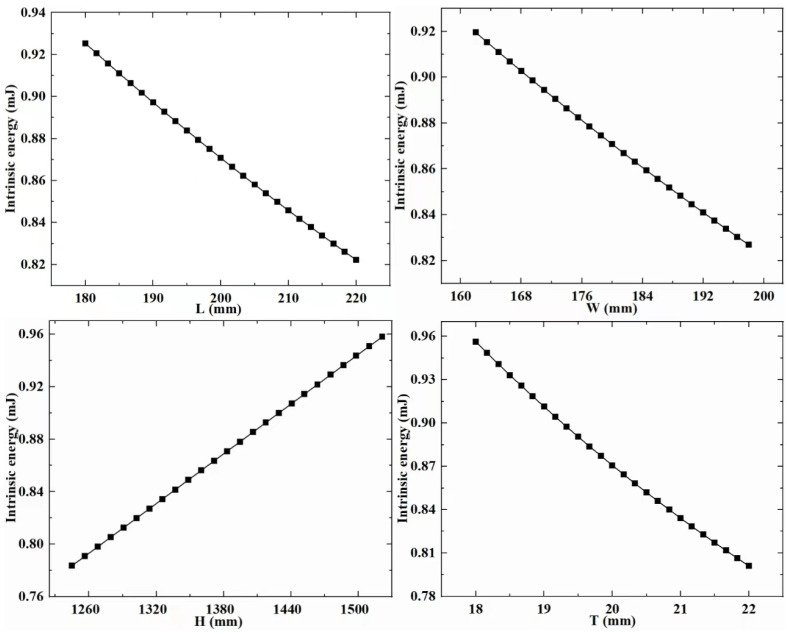
Meta-model c.

RSM is a technique that involves experimental design to collect data and construct visual models. To establish the meta-model in this paper, it is first necessary to extract experimental data for both design variables and response variables. In the above-mentioned study, the design variables of the support column structure were determined. The experimental groups with different design variable combination values were obtained through the CCD experimental design methodology. Subsequently, calculations were performed on these experimental groups to extract data for the response variables that reflect the structural performance of the different experimental groups. Next, the extracted experimental data is used to build a visual meta-model. The meta-model (Figure 6, Figure 7 and Figure 8) is used to explore the relationship between design variables, mass, strain energy, and inherent energy.

Based on the established meta-model, it is observed that the overall trends among the variables in the supporting column structure differ. With increased design variable values, strain energy and inherent energy might decrease, suggesting a nonlinear relationship among the variables. Specifically, for the regularly shaped supporting column structure, as L, W, H, and T increase, the general trend of strain energy decreases from its maximum. In contrast, inherent energy exhibits irregular changes with some increases and decreases, showcasing a disordered variation. It is found that there is a pattern between the design variables, mass, strain energy, and inherent energy. When L, W, and T increase, there is a significant decrease in strain energy and inherent energy. When H increases, strain energy decreases, and inherent energy increase.

### 3.3. Sensitivity Analysis

**Figure 9:**
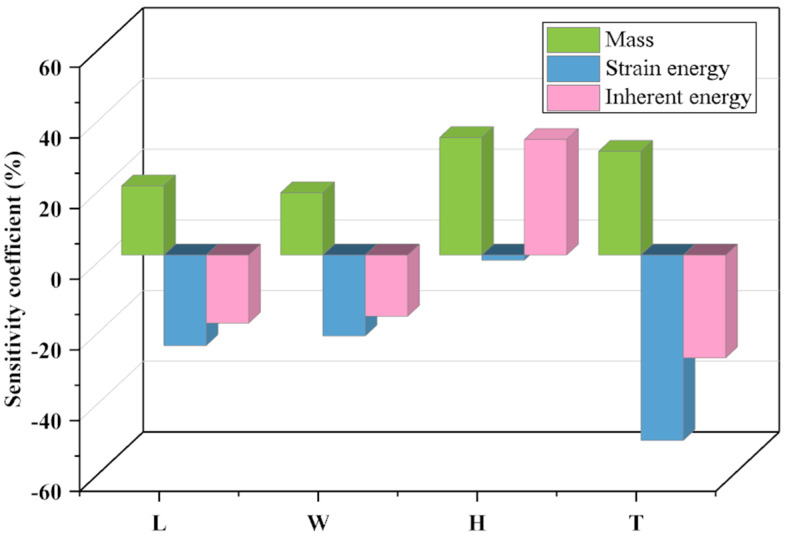
Sensitivity analysis of design variables.

Conduct sensitivity analysis based on the fitted response surface to determine the influence of design variables on the response variables. Through the analysis, sensitivity coefficients for each variable can be obtained, as shown in Figure 9 below:

The sensitivity analysis reveals that for strain energy, variable H has the smallest sensitivity coefficient, while variable T exhibits higher sensitivity coefficients. Looking at inherent energy, a distinctly different situation emerges, where variable H demonstrates a high sensitivity coefficient. Furthermore, it is noteworthy that an increase in sensitivity coefficients leads to a reduction in the design domain. This finding confirms that variable T possesses a smaller design domain in this study, whereas other variables have larger design domains. In summary, the sensitivity analysis results unequivocally indicate that the four variables, L, W, and H, and T, play a crucial role in structural design, providing robust support for the optimization.

## 4. Optimized Design of the NSGA-III Algorithm

### 4.1. Establishment of an Objective Function

The optimization problem of the supporting column structure can be summarized into three aspects: achieving lightweight design, reducing strain energy, and enhancing the inherent energy of the structure. Objective functions were constructed between the design and response variables to accomplish these objectives, utilizing multiple nonlinear regression methods for modeling. This method was employed to simulate the intricate nonlinear relationship among design variables, mass, strain energy, and inherent energy. Mathematical functional expressions depicting the interrelations between various variables can be obtained through nonlinear regression analysis.

(13) $$\begin{array}{l} M=147.9223-0.4339x_{1}-0.4376x_{2}-0.1067x_{3}\\ -6.51780x_{4}-(1.6382\times 10^{-17})x_{1}x_{2}+(3.1446\times 10^{-4})x_{1}x_{3}\\ +0.0218x_{1}x_{4}+(3.1477\times 10^{-4})x_{2}x_{3}+0.021634x_{2}x_{4}\\ +(4.7033\times 10^{-3})x_{3}x_{4}-(4.0213\times 10^{-6})x_{1}{}^{2}+(1.0470\times 10^{-5})x_{2}{}^{2}\\ -(8.4069\times 10^{-8})x_{3}{}^{2}-0.0429x_{4}{}^{2} \end{array}$$

(14) $$\begin{array}{l} S=(8.259\times 10^{-5})-(9.189\times 10^{-8})x_{1}+(4.111\times 10^{-8})x_{2}\\ +(4.118\times 10^{-8})x_{3}-(5.693\times 10^{-6})x_{4}+(5.951\times 10^{-10})x_{1}x_{2}\\ -(4.557\times 10^{-12})x_{1}x_{3}+(4.098\times 10^{-9})x_{1}x_{4}-(5.060\times 10^{-12})x_{2}x_{3}\\ +(5.951\times 10^{-9})x_{2}x_{4}-(4.557\times 10^{-11})x_{3}x_{4}-(4.150\times 10^{-10})x_{1}{}^{2}\\ -(9.755\times 10^{-10})x_{2}{}^{2}-(1.390\times 10^{-11})x_{3}{}^{2}+(5.849\times 10^{-8})x_{4}{}^{2} \end{array}$$

(15) $$\begin{array}{l} I=0.87-0.052x_{1}-0.047x_{2}+0.088x_{3}-0.078x_{4}\\ +(5.494\times 10^{-3})x_{1}x_{2}-(5.187\times 10^{-3})x_{1}x_{3}+(3.967\times 10^{-3})x_{1}x_{4}\\ -(4.668\times 10^{-3})x_{2}x_{3}+(3.567\times 10^{-3})x_{2}x_{4}-(7.760\times 10^{-3})x_{3}x_{4}\\ +(3.026\times 10^{-3})x_{1}{}^{2}+(2.448\times 10^{-3})x_{2}{}^{2}+(1.480\times 10^{-6})x_{3}{}^{2}\\ +(7.892\times 10^{-3})x_{4}{}^{2} \end{array}$$

In the above equation: M is the total mass of the structure, S is the strain energy, I is the inherent energy, and $x_{1},x_{2},x_{3},x_{4}$ denote the design variables.

### 4.2. Multi-Objective Optimization Design

The multi-objective optimization design aims to achieve a lightweight design while satisfying conditions for strain energy and inherent energy. Based on an in-depth analysis of performance indicators, the following multi-objective optimization model is proposed:

(16) $$\min\left\{M,S\right\}\mathrm{and}\max\left\{I\right\}$$

(17) $$s.t.\left\{\begin{array}{l} M_{L}+M_{U}\leq M\\ v^{L}\leq v\leq v^{U}\\ D\left(x\right)\geq 0,W^{L}>0 \end{array}\right.$$

where $v^{L},v^{U}$ are the upper and lower bounds of the variables, $D\left(x\right)$ is the geometric constraints, and $W^{L}$ is the optimized work area. In practical scenarios, the optimized results of the supporting column structure constitute a non-dominated Pareto optimal solution set. This paper integrates the principle of cooperative equilibrium [43] to determine the ultimate optimized frameworks. Pareto optimal solutions are dimensionless.

(18) $${p^{\prime }}_{n}=\frac{p_{n}-k_{pn}}{\sigma_{pn}}$$

where ${p^{\prime }}_{n}$ is the performance index at the Pareto boundary, $k_{pn}$ is the average value, and $\sigma_{pn}$ is the variance of the objective. The minimum point of the desired objective is the ideal point, and the distance from the point on the Pareto solution boundary to the ideal point is:

(19) $$D_{n}=\sqrt{{\left(p_{n}-p_{n,\min}\right)}^{2}}$$

where $p_{n,\min}$ is the minimum over n. The cooperative equilibrium point minimum $D_{n}$ is the Pareto point.

### 4.3. Optimization Results of NSGA-III Algorithm

**Figure 10:**
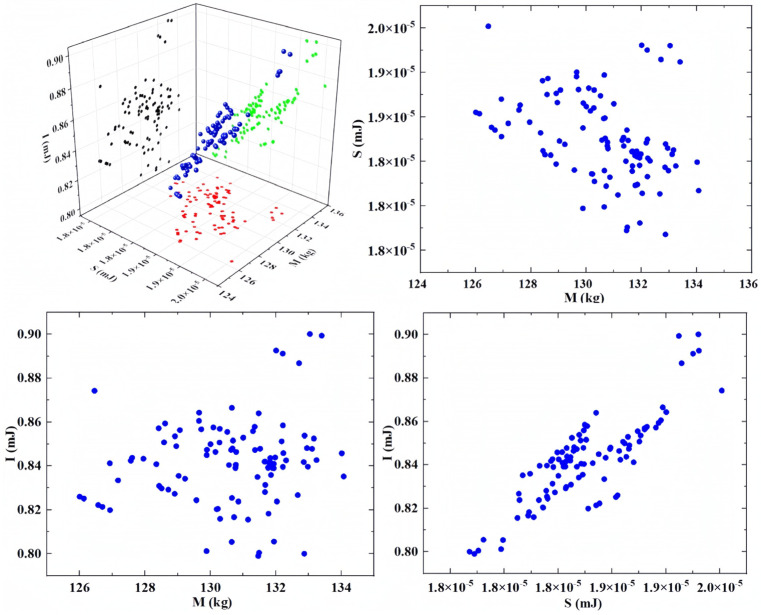
Pareto optimal solution set.

Based on the constructed multi-objective optimization model, MATLAB software was employed to solve the optimization problem using the NSGA-III algorithm. In this process, a population size of 100 was chosen, and 100 generations of optimization computations were performed. The cross-over probability was set to 0.9, and the mutation probability was set to 0.1, ensuring the algorithm’s convergence. From the non-dominated Pareto solution set generated by NSGA-III, the final optimization results were selected using the principle of cooperative equilibrium. The optimized results are shown in Figure 10.

The values of M, S, and I were obtained through the Pareto optimal solution set with specific objective values of 126.46 kg, 2.00 × 10^−5^ mJ, and 0.87 mJ. These objective values were used to constitute the desired values for the optimization results. Next, the cooperative equilibrium principle was used to further define the objective values. The cooperative equilibrium point was defined as the minimum distance from the ideal point to the Pareto boundary. The values of the cooperative equilibrium point for M, S, and I were 132 kg, 1.98 × 10^−5^ mJ, and 0.89 mJ, respectively. The corresponding optimum frameworks of the structure were 183.13 mm in length, 165.05 mm in width, 1324.08 mm in height, and 20.70 mm in thickness.

## 5. Generative Designs Using the Results of NSGA-III Optimization

In this paper, based on the optimal structural framework determined by the principle of synergistic equilibrium, the initial structure of the supporting column is constructed as an experimental object. Subsequently, software iteration is employed to develop the first-generation design structure. The research findings presented above serve as information for the design of the next generation. The genetic algorithm used in the generative design structure employs adaptive selection criteria, encompassing aspects such as structural compliance, topological layout, mass preservation rate, and computational efficiency. During the design phase, a detailed analysis and iterative computations were conducted using ANSYS software to generate an optimal topology structure that satisfies the 0.003 mm displacement constraint. Topology optimization was carried out using the SIMP method [44,45]. Various generative design variables were employed in the experimental process, and the experiments were executed by controlling the mesh adjustment tools in ANSYS. Mesh resolutions ranging from 1 to 9 were used to determine element sizes, transitions, and span angle centers, resulting in coarse meshes of 2000 to 15,000 elements and fine meshes of 16,000 to 80,000 elements. These constraints guided the generative design in an ideal direction. The material parameters of the supporting column structure (Table 2) and the initial parameter set (Table 3) for the generative design are provided as follows:

Generative design’s initial parameter set and response constraints define various structures generated during the software computation process. The generative design process is outlined as follows:

(1) Automatic iterative computations are performed based on the initial parameter set, providing information for the next-generation computations;
(2) Generated structures are evaluated, and tetrahedral meshes are excluded due to their low quality, and the evaluation results are utilized in the next generation;
(3) Model modifications are made based on feedback from the previous optimization algorithm;
(4) Hexahedral meshes are employed for optimization by studying iteratively computed structures;
(5) The quality of different structures is assessed using selected mesh types and design variables;
(6) All generated results are evaluated, and mesh elements with stable structural quality are chosen as the foundation for the next-generation optimization;
(7) Based on the outcomes of extensive iterative computations, the optimal optimization results are selected, accompanied by independent structural solutions;
(8) Trimming and validation are applied to the optimized structure to ensure manufacturing requirements are met.

**Figure 11:**
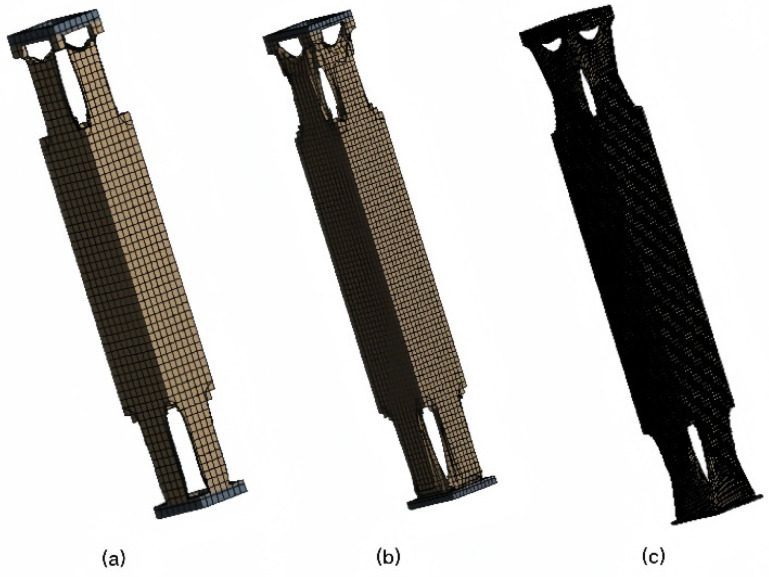
Visualization of preliminary results.

The initial structure of the supporting column, with an overall mass of 132 kg, is designed with a mass preservation rate of 40–50%. The visually generated structure from iterative computations is depicted in Figure 11, and experimental results are presented in Table 4.

The initial experiments above produced three results: a, b and c. From a visual perspective, it can be observed that these results exhibit a hollow layout at both ends, while the central region remains unchanged.

### 5.1. Generative Design of Pre-Optimized Structures

**Figure 12:**
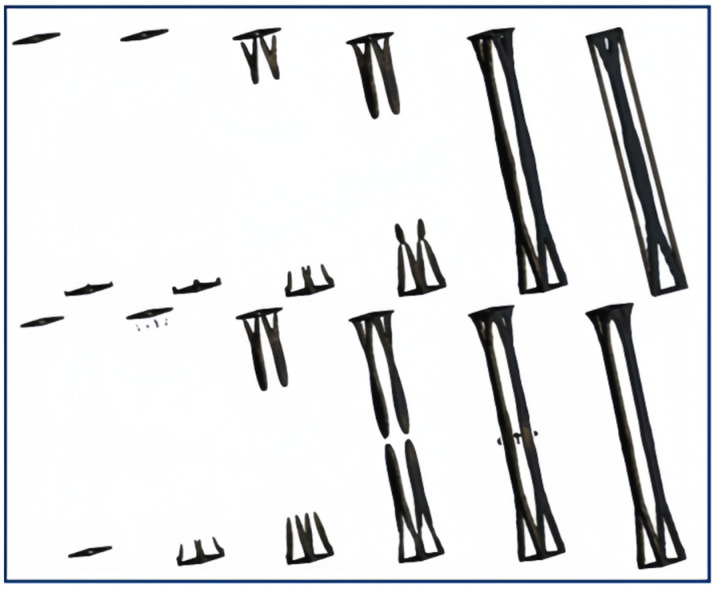
Generative design process for support column structure.

In generative design, the design space is searched under the given design requirements by manual GA algorithms, and individual solution results are continuously modified. The information generated from each structure iteration is passed to the next generation for use, and after generating the structure, the effective structure is output based on the analysis results from the cloud platform. After a large number of variable adjustments and combinations of different generations, the pre-solution of the generative design is obtained. The generative design process of the structure is shown in Figure 12.

**Figure 13:**
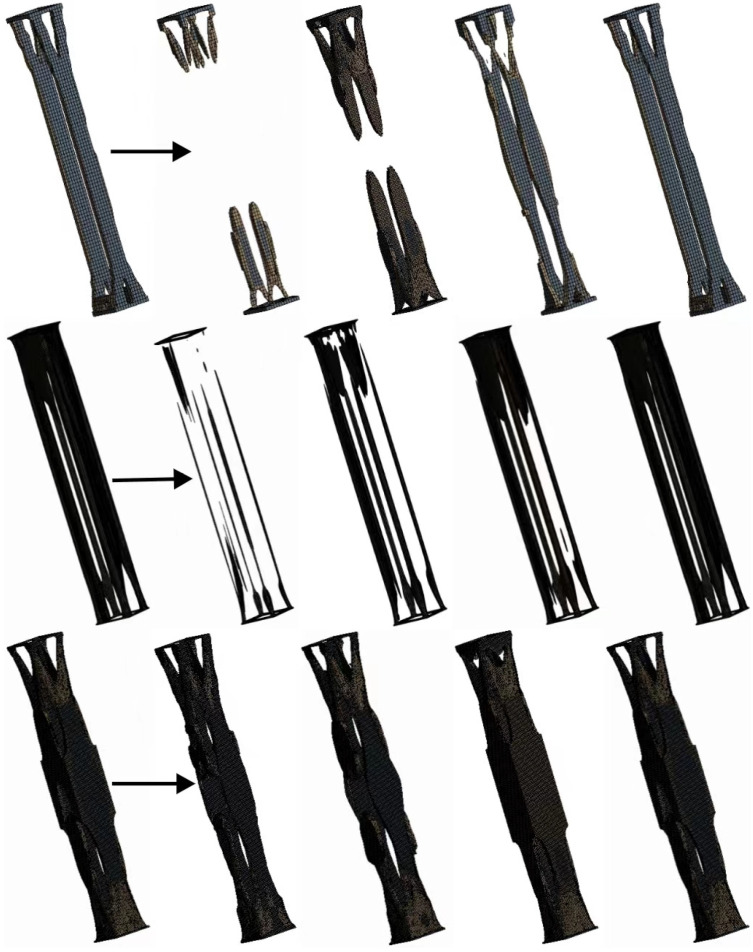
Generative design process for preliminary results.

The research has revealed that the evolutionary process of the supporting column structure is carried out symmetrically on the sides, tending towards accumulating and evolving towards the central region. Therefore, in the next generation of computations, structural symmetry will be employed as a constraint in design. In terms of mesh type, a hexahedral mesh is selected. Generative design is a complex process that does not follow a linear response. Due to this characteristic, linear methods are not applicable, thus resorting to the manual genetic algorithm approach to attain an optimized structure for the supporting columns. The preliminary results of the generative structure are illustrated in Figure 13.

Figure 11 provides a clear illustration of the generative design process. Within this diagram, the significance of incorporating an initial parameter set and control methods in the generative design process is emphasized. By precisely defining initial parameters and constraint conditions, the achievement of structural design. Following extensive generations of optimization iterations, the solution for the generative design structure has been established: (1) utilization of a hexahedral mesh type; (2) imposition of symmetry constraints on the structure; (3) selection of a mass retention rate ranging from 40% to 55%; and (4) consideration of other specified conditions, as indicated in Table 3.

### 5.2. Determining Mesh Elements for Generative Design

Perform parametric static analysis on the supporting column structure, investigating the relationships among different elements and nodes of the mesh. Evaluate the mesh elements that support the column structure and obtain configurations with high fitting accuracy between the two to obtain an accurate optimized structure. Furthermore, elements with sizes related to structural stress and location can be achieved through a localized mesh control and refinement procedure. In this study, the convergence criterion for the mesh is set to achieve a convergence rate of 5% to meet the results’ accuracy requirements. During the generative design phase of the supporting column, numerous different structures were generated. However, these structures were not adopted to balance computational efficiency and reduce complexity. A more in-depth investigation of the optimization process was conducted to enhance structural quality further. The outcome of the generative design in this study is presented in Table 5.

**Figure 14:**
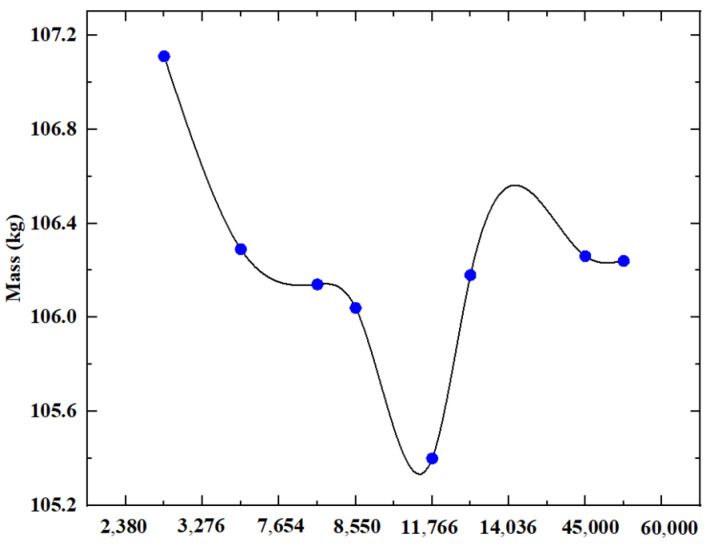
Structural mass produced by different mesh elements.

Simultaneously, geometric shapes that could not be modified in any way were excluded. Under consistent constraint conditions, varying mesh elements yield differing quality. During the generative design phase, the structural quality resulting from the generation of diverse mesh elements is depicted in Figure 14.

As depicted in Figure 12, the *X*-axis denotes an incremental increase in mesh elements from left to right. It becomes evident that the quality of the supporting column structure’s grid units stabilizes around 50,000, prompting the exclusion of alternative mesh element configurations. The results of the structural grid analysis are presented in Table 6.

### 5.3. Optimization Results of Support Column Components

**Figure 15:**
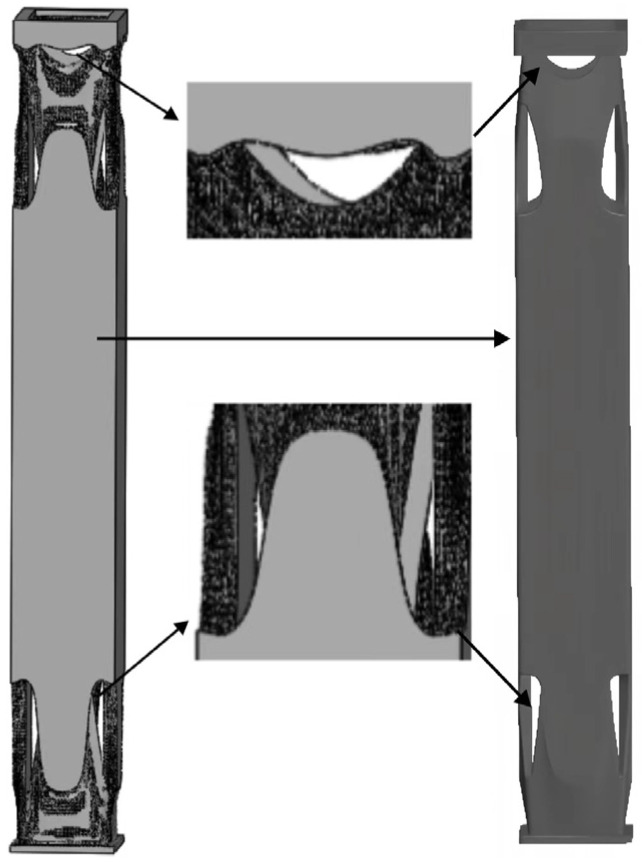
Processing of the final generative optimized design structure.

Based on the feedback from the different generations of the algorithm, it was determined that the structure with a final mass of 106.24 kg was obtained using mesh type, mesh elements, mass retention, and symmetric design constraints. However, due to the presence of numerous burrs and facets on the generated parts, this poses high computational efficiency and resource demands. Therefore, after exploring various methods and considering different commercial software, the simplification of the irregular structural planes was pursued. After multiple trials, the Creo tool was employed to process the structures created by ANSYS’s generative design. The simplification followed these rules: addressing sharp edges of components, addressing transitional connected facets, and handling irregular geometric shapes. The resultant optimized structure quality amounted to 107.65 kg (Figure 15).

### 5.4. Verification Results of Optimized Design Structures

The framework with optimal support column structure is obtained by the NSGA-III algorithm. Subsequently, a generative design of this frame was carried out to obtain the optimized structure, and at the same time, the feasibility of the structure was verified. The results showed that the mass of the support column was reduced to 107.65 kg, the strain energy was 7.30 × 10^−4^ mJ, and the inherent energy of the structure reached 1.70 mJ. The mass, strain energy, and inherent energy of the initial structure (IS), the structure after NSGA-III optimization (NOS), and the comprehensive and optimized design structure (CODS) are presented in Table 7. Under identical load and constraint conditions, the experimental data reveal that the mass of the supporting column structure decreased by 27%. Simultaneously, the strain energy meets the design requirements (well below 4.8 mJ), while the inherent energy of the structure increased by 0.95 times, ensuring the stiffness performance of the supporting column structure.

**Figure 16:**
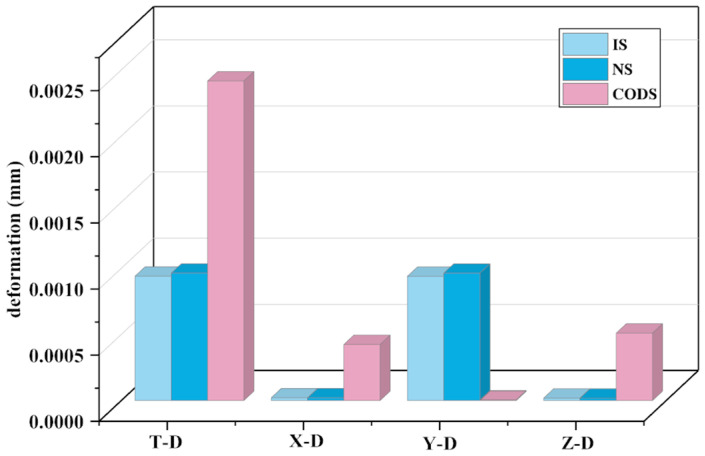
Deformation of the structure.

Upon validation, it was observed that the structure optimized by NSGA-III experienced an 11% reduction in mass while the inherent energy changed a little. The framework of the structure has little effect on the inherent energy. After undergoing comprehensive optimization, a noticeable change in inherent energy occurred, signifying that the structural layout significantly affects inherent energy. The deformation of the overall structure is depicted in Figure 16.

**Figure 17:**
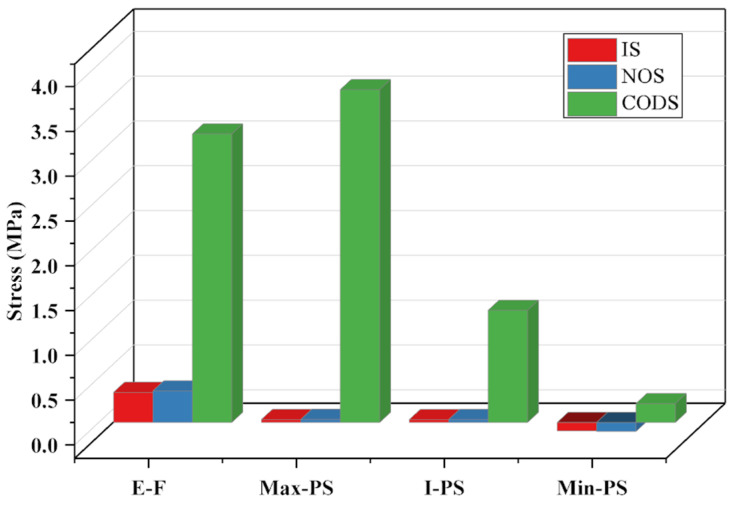
The stress situation of the structure.

In Figure 16, T-D represents the overall deformation of the structure, X-D indicates the deformation along the *X*-axis, Y-D signifies the deformation along the *Y*-axis, and Z-D represents the deformation along the *Z*-axis. Research findings indicate that, under the same applied load on the components, as the structure’s mass decreases, its deformation increases. This aligns with a common trend and validates the correctness of the research direction. Along the X, Y, and Z axes, the deformation of the comprehensively optimized structure is 0.00051 mm along the *Z*-axis, with the smallest deformation occurring along the X and Y axes. The deformation of the initial structure and the NSGA-III optimized structure remain largely consistent. Although all three structures meet the usage requirements, the comprehensively optimized structure not only boasts the lightest mass but also possesses the requisite flexural strength. Furthermore, the deformation of the comprehensively optimized structure is 0.0024 mm, which is below 0.003 mm, thus substantiating the efficacy of the comprehensive optimization design structure. The stress situation of the structure is illustrated in Figure 17.

To further validate the feasibility of the proposed method, stress verification of the structure was conducted. In Figure 15, E-F represents equivalent stress, Max-PS indicates the maximum principal stress, I-PS denotes the intermediate principal stress, and Min-PS stands for the minimum principal stress. The results are as follows: (1) the stress levels of the initial structure and the structure optimized using the NSGA-III algorithm are nearly identical, exhibiting minimal fluctuations; (2) among the three structures, the maximum stress values all remain below the yield strength, affirming the viability of these structures; and (3) the comprehensive optimization approach effectively reduces part mass by 27% and increases inherent energy by 0.95 times. Therefore, the comprehensive optimization design method has great advantages.

The validation of the above research results shows that the proposed integrated optimization design method not only reduces the mass of the structure, but also effectively improves the performance of the structure. At the same time, the effectiveness of the method is also more comprehensively verified by the stresses and strains calculated by simulation. This study opens up a new research method for the field of mechanical structure optimization.

## 6. Discussion

In this study, an integrated optimization design method is proposed by integrating the intelligent optimization algorithm with generative design. The method successfully solves the problem of lightweight design of structures involving multi-variables and multi-objectives. It fills the gap between the NSGA-III algorithm and generative design in structural optimization. By adopting the integrated optimization design method, a lightweight design can be realized, and the structural performance can be improved. At the same time, diversified design requirements are satisfied. It opens up new avenues for the lightweight design of mechanical structures and has excellent potential for continued improvement.

To further enhance the integrated optimization design method, a comparative study of different crossover, variant, and population settings of NSGA-III can be considered to find the best configuration for the fastest convergence. Meanwhile, some steps in generative design currently require manual operation. In the future, the automatic generation of design structures using Autodesk Inventor software can be considered to improve the efficiency of the integrated optimization method. In addition, it is essential to acknowledge that this study performed a comprehensive optimization design for a simple structure. Future research will apply the integrated optimal design method to complex structures and further refine the method by studying different types of structures to develop an integrated optimal design method with a broader range of applicability.

## 7. Conclusions

In the past, mechanical structures were optimized using separate methods, such as structural dimension adjustments and topology optimization. Furthermore, most research overlooked the stiffness optimization of the structure and did not thoroughly investigate the relationships between design variables, quality, strain energy, and inherent energy. In this paper, a comprehensive optimization design method is proposed by combining the NSGA-III algorithm with generative design. The method is verified in the structural optimization of support columns, which opens up a new research direction for the development of lightweight mechanical structures. The main research achievements are as follows:

(1) This paper proposes a comprehensive optimal design methodology, innovatively integrating NSGA-III and generative design into structural optimization to solve the multivariate and multi-objective structural lightweight design problem.

(2) A meta-model of each component was developed, and the relationships between design variables and mass, strain energy, and inherent energy were obtained. In addition, a sensitivity analysis of the design variables was performed to demonstrate the importance of the design variables.

(3) The optimization objective function is established based on multivariate nonlinear regression, and the NSGA-III algorithm is applied to obtain the optimal structural frame, which lays the foundation for the comprehensive optimization design.

(4) The experimental results of the integrated optimization design show that the method reduces the structural mass by 27% and increases the inherent energy by 0.95 times. At the same time, strain and stress meet the usage requirements.

In the future, the proposed method will be implemented to manufacture the supporting column structure in the factory. Then, the study of the overall performance of the dual robot gripper unloading device is continued to further explore the optimized design of the framework and structural layout of the support column components.

## Figures and Tables

**Table 1:** Experimental groups for support column component.

Std	Design Variable 1 L: Length (mm)	Design Variable 2 W: Width (mm)	Design Variable 3 H: Highly (mm)	Design Variable 4 T: Thickness (mm)
1	200.00	180.00	1383.00	20.00
2	180.00	180.00	1383.00	20.00
3	220.00	180.00	1383.00	20.00
4	200.00	162.00	1383.00	20.00
5	200.00	198.00	1383.00	20.00
6	200.00	180.00	1244.70	20.00
7	200.00	180.00	1521.30	20.00
8	200.00	180.00	1383.00	18.00
9	200.00	180.00	1383.00	22.00
10	185.92	167.32	1285.60	18.59
11	214.08	167.32	1285.60	18.59
12	185.92	192.68	1285.60	18.59
13	214.08	192.68	1285.60	18.59
14	185.92	167.32	1480.40	18.59
15	214.08	167.32	1480.40	18.59
16	185.92	192.68	1480.40	18.59
17	214.08	192.68	1480.40	18.59
18	185.92	167.32	1285.60	21.41
19	214.08	167.32	1285.60	21.41
20	185.92	192.68	1285.60	21.41
21	214.08	192.68	1285.60	21.41
22	185.92	167.32	1480.40	21.41
23	214.08	167.32	1480.40	21.41
24	185.92	192.68	1480.40	21.41
25	214.08	192.68	1480.40	21.41

**Table 2:** Material properties of support column structures.

Material	Density/(kg/m³)	Young’s Modulus/10^11^ Pa	Poisson’s Ratio	CTE/C^−1^
Structural steel	7850	2.1	0.3	1.2 × 10^−5^

**Table 3:** The initial set of parameters for the generative design.

Design Variables	
Mesh type	Tetrahedron, hexahedron
Structural parameters	L:183.13 W: 165.05 H:1324.08 T: 20.70 unit: (mm)
Design quality of structures	Less than 132 kg
Mesh unit	2000–80,000
Transition	Fast, slow
Span angle center	Large angle 60~91°, medium 24~75°, fine 12~36°
Mesh convergence rate	5%
Iterative computation	10–500
Displacement constraint	0.003 mm
Response constraints	Quality retention range 40–60%

**Table 4:** Experimental results.

Variable	a	b	c
Transition	Fast		
Span angle center	Large angle		
Iterative computation	10–500		
Mesh	Hexahedral coarse mesh	Hexahedral transition mesh	Hexahedral fine mesh
Mass	107.11 kg	106.29 kg	106.24 kg

**Table 5:** Settings for the generative optimization process.

A	B	C	D
Fine mesh	Coarse mesh	Fine mesh	Fine mesh
Quality retention rate (50%)	Quality retention rate (50%)	Quality retention rate (40–55%)	Quality retention rate (40–50%)
Design constraints	Structural symmetry		
No generative limiting displacement constraints	Generative limit displacement constraint (0.003 mm)	No generative limiting displacement constraints	Generative limit displacement constraint (0.003 mm)
Support column mass 106.26 kg	Support column mass 106.8 kg	Support column mass 130.94 kg	Support column mass 106.24 kg

**Table 6:** Results of the mesh study.

Mesh Settings	
Minimum element size	0.400
Evaluation factor	0.998
Maximum corner angle	92
Number of elements	51,300
Number of nodes	257,490

**Table 7:** Structural properties of support columns.

Typology	Mass (kg)	Strain Energy (mJ)	Inherent Energy (mJ)
IS	147.65	1.85 × 10^−5^	0.87
NOS	132.00	1.98 × 10^−5^	0.89
CODS	107.65	7.30 × 10^−4^	1.70

## Data Availability

Important data are contained within the article. Additional data may be available upon reasonable request to the corresponding author.
